# Study on Quality Response to Environmental Factors and Geographical Traceability of Wild *Gentiana rigescen*s Franch

**DOI:** 10.3389/fpls.2020.01128

**Published:** 2020-07-22

**Authors:** Lu Liu, Zhi-tian Zuo, Fu-rong Xu, Yuan-zhong Wang

**Affiliations:** ^1^ College of Traditional Chinese Medicine, Yunnan University of Chinese Medicine, Kunming, China; ^2^ Institute of Medicinal Plants, Yunnan Academy of Agricultural Sciences, Kunming, China

**Keywords:** *Gentiana rigescens*, environmental factors, iridoid content, quantitative determination, Fourier transform infrared, quality control, geographical traceability

## Abstract

*Gentiana rigescens* Franch. ex Hemsl. is an important medicinal plant in China and the over exploitation of wild resources has affected its quality and clinical efficacy. The accumulation of plant secondary metabolites is not only determined by their genetic characteristics but also influenced by environmental factors. At present, many studies on evaluating the environmental conditions of its planting area are still in the qualitative stage. Therefore, it is necessary to establish a systematic evaluation method to deeply analyze the impact of environmental factors on the quality of medicinal materials and quickly verify the geographical origin. In this study, the contents of five iridoids (loganic acid, swertiamarin, sweroside, gentiopicroside and 6'-*O*-β-D-glucopyranosylgentiopicroside) of *G. rigescens* from 45 different origins (including 441 individuals) of Yunnan Province in China were analyzed by high performance liquid chromatography. Analytical procedures of one-way analysis of variance, correlation analysis, principal components analysis, and hierarchical cluster analysis were employed to interpret the correlation of iridoid content and environmental factors. Fourier transform infrared spectroscopy (FT-IR) combined with two multivariate analysis methods (partial least squares discriminant analysis; support vector machines, SVM) was used to discriminate four major producing areas (158 individuals). The combination of SVM with grid search algorithm achieved an accuracy of 100% in the test set. One-way analysis of variance showed that the contents of five iridoids in root tissues of *G. rigescens* varied significantly among different origins, which was also verified by the chemometrics analysis results of hierarchical cluster analysis. The results of correlation analysis indicated that the high value of altitude and precipitation were unfavorable for the accumulation of these five iridoids. A correlation between increase of temperature and iridoid accumulation was observed. This study provided a certain theoretical basis for the resource protection and development of *G. rigescens* based on the correlation analysis between the ecological environment factors and quality.

## Introduction

Traditional Chinese medicine (TCM) known as Radix Gentianae, with root and rhizome being the major parts used medicinally. The genuine producing area, which refers to the terminology of *daodi* in Chinese, of *Gentiana rigescens* Franch is located in Yunnan Province of China, and medicines produced in this place have higher quality and superior clinical effects than the same species originated from other regions (Zhao et al., 2012). The iridoids gentiopicroside (GE), swertiamarin (ST), and sweroside (SW) are the main active ingredients of *G. rigescens. G. rigescens* has liver-protecting (Xu et al., 2013), cholagogic (Lim et al., 2007), anti-inflammatory (Öztürk et al., 2006; Wang et al., 2013), and anti-oxidant (Jaishree and Badami, 2010) activities, which has long been used in treating inflammation, damp-heat, jaundice, eczema, and itching (National Pharmacopoeia Committee, 2015; Chu et al., 2016). In recent years, the root of Radix Gentiana is found to contain the 2,3-dihydroxybenzoates (gentisides A-B) with favorable neurotrophic and protective effects, making it a potential material as the functional food against Alzheimer's disease (AD) (Gao et al., 2010; Mustafa et al., 2016). As an important natural medicine, *G. rigescens* is used as a raw material of Chinese drug such as “Long Dan Xie Gan Tang,” but also in clinical medicine. Hence, *G. rigescens* is a kind of plants with remarkable physiological activity, which has shown great value in the development and utilization of medicinal plant resources.

With the deepening of study in recent years, the annual demand for *G. rigescens* in clinical and health care product industries is up to 40–60 tons (Wang et al., 2017), and the related quality standard is also improved. As a result, the wild resources can no longer meet the increasing demand for *G. rigescens*, which may cause resource depletion problems (Wang et al., 2017). At present, progresses have been made in the artificial cultivation technology to protect the original plants from extinction due to the growing demands and excessive harvesting (Tang et al., 2013). However, the blind introduction without considering the ecological suitability also gives rise to a series of problems, such as the uneven quality of medicinal materials produced across different origins and the seriously excessive heavy metals in medicinal materials. Therefore, it is necessary to further examine the quality and trace the geographical origin of wild *G. rigescens*. Vibration spectroscopy technology, including the Fourier transform infrared spectroscopy (FT-IR), is a convenient quality evaluation approach, which has been widely adopted due to its advantages of rapidness and non-invasiveness (Ma et al., 2018). Moreover, the obtained spectra reflect all sample chemical information compared with that of high performance liquid chromatograph (HPLC) data. Generally, multivariate analysis is utilized to mine the spectral information for better predict the real geographical origin. Besides, the linear partial least squares discriminant analysis (PLS-DA) and nonlinear support vector machine (SVM) have been extensively used.

The active ingredients of medicinal plants are important indexes to evaluate the quality of Chinese medicinal materials (Wu et al., 2018). For a majority of plants, the accumulation of secondary metabolites is determined not only by their own genetic characteristics, but also by the ecological environmental factors such as light, water, temperature, and soil moisture (Kawabata et al., 2001; Wu et al., 2014). Plants will determine the species and quantity of secondary metabolites synthesized based on the changes in their own environment (Linhart and Grant, 1996). This can be ascribed to the combined consequence of long-term adaptation and selection to complex environments in plants during the process of evolution, and the environmental choices for plants (Ackerly et al., 2000). As the main producing area of *G. rigescens*, Yunnan Province has a special geographical environment of low-latitude plateau, and there are various climate types in the distribution area, from north subtropical to south subtropical and marginal tropics (He and Liu, 2001; Zheng et al., 2015). At present, many studies have focused on exploring the differences and similarities of medicinal materials collected from various climate zones, which ignore the influence of environmental factors on the diversity of medicinal plants (Assogbadjo et al., 2006; Seidel et al., 2008). In addition, other factors, including the accumulated temperature and extreme temperature that are monitored and detected in different climate zones of Yunnan Province, are often ignored. Therefore, efforts should be made to explore the connection between environmental factors and the medicinal material. It is of great significance to improve the quality of medicinal materials, standardize the production of *G. rigescens* medicinal plants and protect the wild resources (Huang et al., 2012).

This study aimed to demonstrate the feasibility of distinguishing the geographical origin of *G. rigescens* based on the FT-IR spectral information using the PLS-DA and SVM algorithms. The contents of main indicator components were compared in wild *G. rigescens* from different origins in combination with the ecological environmental factors, so as to illustrate the correlations of quality of medicinal materials with the ecological environmental factors. Moreover, the effects of seven environmental factors on the contents of five iridoids in the root tissues of *G. rigescens* were investigated through statistical analysis. Besides, principal component analysis (PCA) and correlation analysis (CA) were adopted to reveal the relevance and dependency between environmental factors and medicinal materials. One-way analysis of variance (ANOVA) and hierarchical cluster analysis (HCA) were also performed to explore the similarity of iridoid chemical profiles monitored in the samples. Findings in this study can not only provide a deeper insight into the resource assessment of *G. rigescens*, but also serve as a reference for the selection and breeding of excellent crops of this species.

## Materials and Methods

### Plant Materials and Chemical Reagent

**Figure 1:**
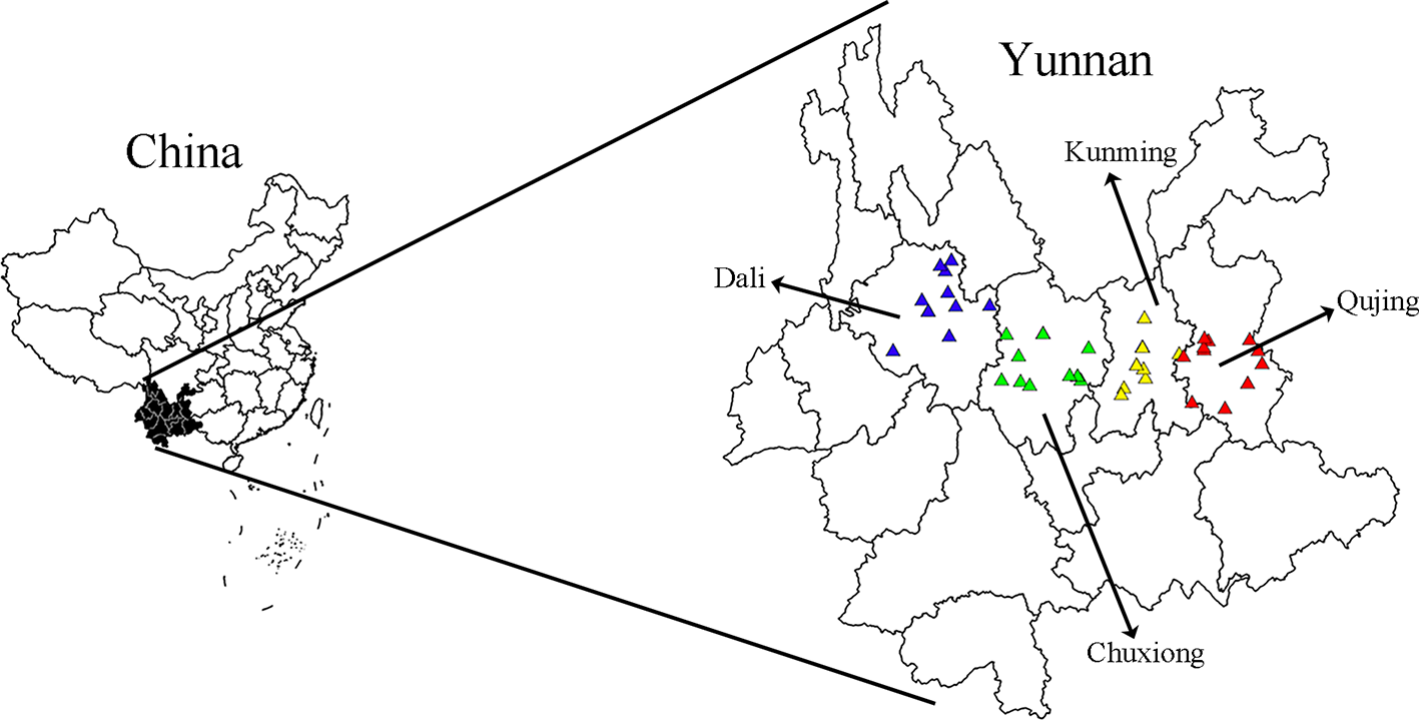
The geographical location of *Gentiana rigescens* samples.

The wild *G. rigescens* samples in the blooming period were obtained from four prefecture-level cities in Yunnan Province, southwestern China. Under the premise of ensuring sustainable utilization and representative, 45 batch samples (441 individuals) were collected and 5–11 individuals were covered in each batch as independent biological replicates. Among them, 158 individuals of 16 batch samples (No.1, 3, 7, 11, 12, 14, 15, 17, 23–26, 35–38) were used for geographical traceability research. All samples were identified as *Gentiana rigescens* Franch. ex Hemsl. by Professor Hang Jin (Institute of Medicinal Plants, Yunnan Academy of Agricultural Sciences). The detailed sample information was shown in **Figure 1** and **Table 1**. All root tissues of *G. rigescens* were dried at 50°C to consistent weight individually by electric thermostatic drying oven (Experimental Instrument Factory, Shanghai, China). Each dried sample was pulverized by DFY-500 kibbler (Wenling City Forest Machinery, Zhejiang, China) and sifted by a 60-mesh sieve. The fine powders were stored in sealed packets at room temperature until further analysis.

**Table 1:** The geographical location and habitat situation of *Gentiana rigescens* sample.

No.	Location	Harvest time	Sample size	Longitude	Latitude	Altitude (m)	Average annual temperature (°C)	5–7 monthly mean temperature (°C)	Average annual precipitation (mm)	5–10 monthly mean precipitation (mm)
1	Guandu Kunming	2013.01.07	10	E102^°^54'48.41”	N25^°^11'33.46”	2,127	15.5	19.9	176.4	142.7
2	Songming Kunming	2013.01.07	9	E102^°^54'53.33”	N25^°^12'31.66”	2,216	14.4	19.2	141.5	146.7
3	Xundian Kunming	2013.01.07	11	E102^°^44'53.76”	N25^°^20'16.61”	2,195	15.5	19.9	176.4	142.7
4	Wuhua Kunming	2013.01.12	10	E102^°^37'55.66”	N25^°^04'17.55”	2,091	15.5	19.9	176.4	142.7
5	Xishan Kunming	2013.01.12	10	E102^°^32'43.35”	N25^°^04'34.02”	2,134	15.5	19.9	176.4	142.7
6	Songming Kunming	2013.01.23	10	E102^°^58'27.98”	N25^°^26'02.44”	2,509	14.4	19.2	141.5	146.7
7	Xundian Kunming	2013.01.23	8	E102^°^51'43.64”	N25^°^33'12.62”	1,825	15.5	19.9	176.4	142.7
8	Xundian Kunming	2013.01.24	10	E102^°^25'19.49”	N25^°^28'01.71”	1,919	15.5	19.9	176.4	142.7
9	Xundian Kunming	2013.01.03	10	E102^°^51'44.03”	N25^°^33'18.64”	1,826	15.5	19.9	176.4	142.7
10	Songming Kunming	2013.01.03	5	E102^°^46'12.58”	N25^°^19'33.25”	2,186	14.4	19.2	141.5	146.7
11	Luquan Kunming	2013.01.04	7	E102^°^52'37.31”	N25^°^54'18.49”	2,089	15.5	19.9	176.4	142.7
12	Bingchuan Dali	2012.11.24	10	E100^°^22'49.56”	N25^°^56'54.17”	2,076	18.4	23.9	86.1	87.2
13	Eryuan Dali	2012.11.24	9	E99^°^56'06.78”	N25^°^59'40.42”	2,159	14.4	19.7	98.6	107.1
14	Heqing Dali	2012.11.24	10	E100^°^17'42.83”	N26^°^29'48.55”	2,396	13.8	19.0	105.1	158.2
15	Yongping Dali	2012.12.18	10	E99^°^41'31.89”	N26^°^29'48.86”	2,851	15.8	21.0	145.8	138.1
16	Xizhou Dali	2013.01.30	9	E99^°^41'30.14”	N25^°^53'12.82”	2,321	15.1	19.7	146.3	149.1
17	Heqing Dali	2013.01.30	10	E99^°^41'30.96”	N26^°^04'54.29”	2,608	13.8	19.0	105.1	158.2
18	Heqing Dali	2013.01.30	10	E99^°^41'30.93”	N26^°^21'35.78”	2,698	13.8	19.0	105.1	158.2
19	Heqing Dali	2013.01.30	10	E99^°^41'30.04”	N26^°^25'28.22”	2,822	13.8	19.0	105.1	158.2
20	Fengyi Dali	2013.01.30	10	E99^°^43'37.47”	N25^°^34'29.99”	2,231	15.1	19.7	146.3	149.1
21	Bingchuan Dali	2013.02.15	10	E103^°^17'42.26”	N25^°^58'17.21”	2,046	18.4	24.0	86.1	87.2
22	Xizhou Dali	2013.01.24	9	E103^°^17'39.74”	N25^°^53'07.70”	2,652	15.1	19.7	146.3	149.1
23	Luoping Qujing	2012.12.01	9	E100^°^50'24.18”	N25^°^11'18.61”	1,541	15.3	20.7	199.9	230.6
24	Zhanyi Qujing	2012.12.18	9	E100^°^50'26.34”	N25^°^41'10.07”	2,705	15.1	19.6	134.2	131.3
25	Fuyuan Qujing	2012.12.28	10	E100^°^50'27.73”	N25^°^23'20.55”	2,300	14.3	19.4	137.1	151.0
26	Fuyuan Qujing	2012.12.29	10	E100^°^50'28.26”	N25^°^42'41.82”	2,565	14.3	19.4	137.1	151.0
27	Malong Qujing	2013.01.23	8	E100^°^50'30.70”	N25^°^27'32.30”	2,025	13.8	18.2	142.0	137.3
28	Malong Qujing	2013.01.23	9	E100^°^50'34.84”	N25^°^28'53.84”	1,945	13.8	18.2	142.0	137.3
29	Zhanyi Qujing	2013.01.23	10	E100^°^50'35.29”	N25^°^38'55.62”	2,485	15.1	19.6	134.2	131.3
30	Zhanyi Qujing	2013.01.23	10	E100^°^50'35.02”	N25^°^45'04.67”	1,895	15.1	19.6	134.2	131.3
31	Fuyuan Qujing	2013.01.23	10	E100^°^50'35.54”	N25^°^29'43.97”	2,088	14.3	19.4	137.1	151.0
32	Shizong Qujing	2013.01.23	9	E100^°^50'36.29”	N24^°^50'25.46”	2,315	14.3	19.1	159.8	162.4
33	Luliang Qujing	2013.01.23	10	E103^°^31'16.50”	N24^°^49'00.30”	2,044	15.1	19.6	134.2	131.3
34	Wuding Chuxiong	2013.01.16	11	E105^°^00'00.03”	N25^°^31'46.32”	1,576	15.2	20.5	113.0	142.0
35	Dayao Chuxiong	2013.01.16	10	E105^°^04'11.78”	N25^°^40'18.26”	1,337	15.8	20.5	127.4	124.3
36	Nanhua Chuxiong	2013.01.16	10	E105^°^04'12.16”	N25^°^06'01.29”	1,371	14.8	20.0	125.8	124.6
37	Zixi Chuxiong	2013.01.16	10	E105^°^04'15.14”	N25^°^01'25.15”	1,344	16.2	21.1	150.2	131.7
38	Lufeng Chuxiong	2013.01.16	10	E105^°^26'43.97”	N25^°^11'20.26”	1,315	16.2	21.4	133.5	137.2
39	Nanhua Chuxiong	2013.01.12	9	E105^°^26'50.41”	N25^°^09'26.27”	1,366	14.8	20.0	125.8	124.6
40	Yaoan Chuxiong	2013.01.13	10	E101^°^23'25.76”	N25^°^38'54.35”	1,973	15.5	20.4	114.2	115.9
41	Zixi Chuxiong	2013.01.12	10	E101^°^23'25.76”	N25^°^09'26.39”	1,911	16.2	21.1	150.2	131.7
42	Lufeng Chuxiong	2013.01.12	10	E101^°^51'58.47”	N25^°^38'58.80”	1,169	16.2	21.4	133.5	137.2
43	Dayao Chuxiong	2013.01.12	10	E101^°^51'58.09”	N25^°^39'59.60”	1,160	15.8	20.5	127.4	124.3
44	Yaoan Chuxiong	2013.01.12	10	E101^°^51'58.26”	N25^°^24'02.50”	2,078	15.5	20.4	114.2	115.9
45	Lufeng Chuxiong	2013.01.12	10	E101^°^55'14.08”	N25^°^24'03.02”	2,598	16.2	21.4	133.5	137.2

**Figure 2:**
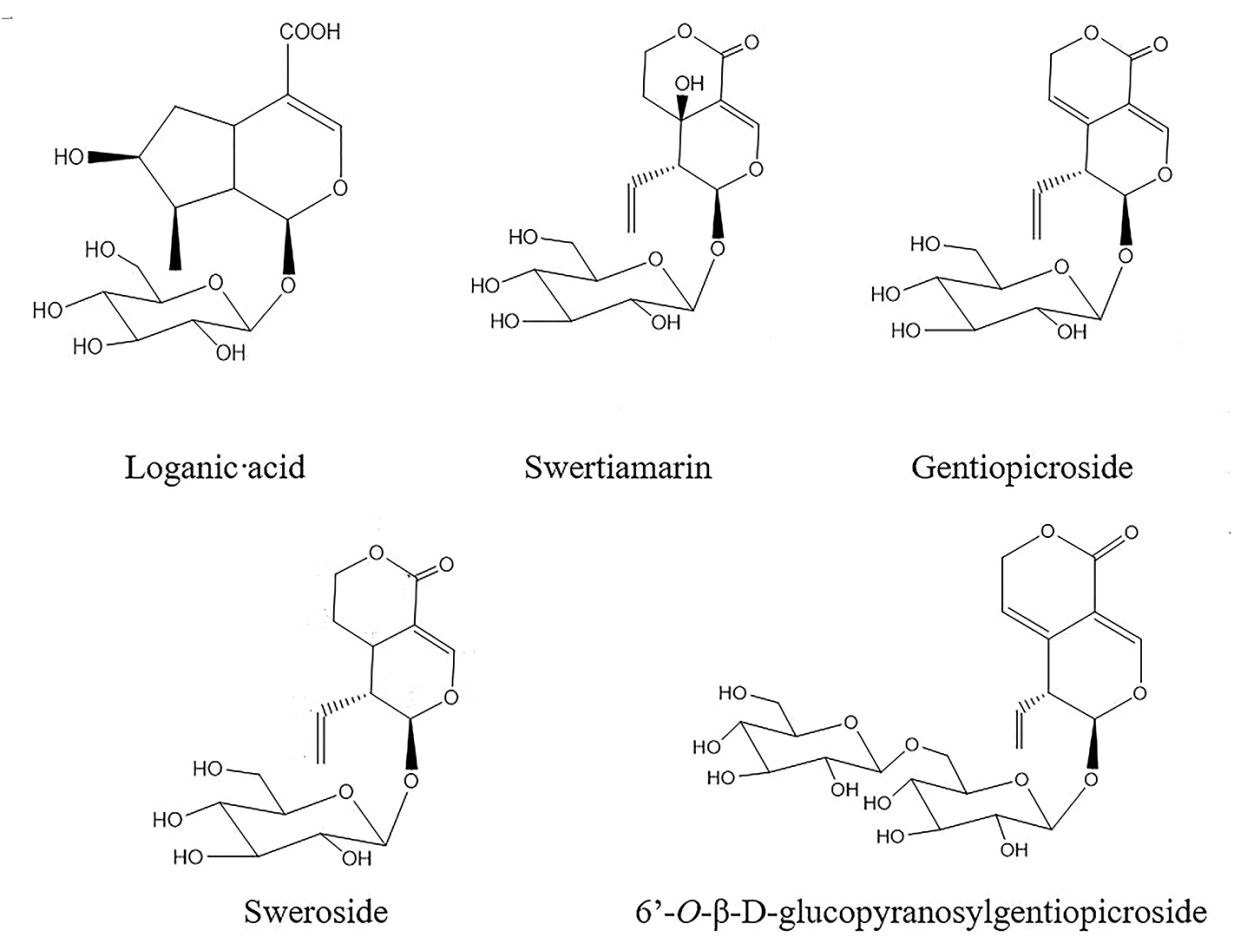
Chemical structures of five iridoids in root tissue of *Gentiana rigescens*.

The methanol and acetonitrile of HPLC grade were purchased from Thermo Fisher Scientific (Massachusetts, USA). The formic acid of HPLC grade was provided by DikmaPure (Beijing, China). Analytical grade methanol reagent used for extraction was purchased from Xilong Chemical Company, Ltd. (Chengdu, China). The ultra-pure water that was injected into the HPLC system was purified by a UPTL-II-40L system (Chengdu, China). The reference compounds of loganic acid (LA) and GE were provided by the China Institute for Food and Drug Control (Beijing, China). The 6'-*O*-β-D-glucopyranosylgentiopicroside (GG) was provided by Shanghai Yihe Biological Technology (Shanghai, China). SW and swertiamain were purchased from Shanghai Shifeng Biotechnology (Shanghai, China). All reference compounds were analyzed by HPLC analysis with the purity of at least 98% and the structures were shown in **Figure 2**.

### Infrared Spectra Acquisition and Preprocessing

A total of 158 dried root powders were analyzed by the Fourier transform infrared spectrometer (FT-IR, Perkin Elmer, USA) equipped with the deuterated triglycine sulfate (DTGS) detector. KBr pellets were made by mixing 1 mg of sample and 100 mg of spectrometry grade KBr for further detection. FT-IR spectra were recorded by 16 co-added scans in the wavenumber range of 4,000–400 cm^-1^ with a resolution of 4 cm^-1^. Each sample was scanned three times in parallel, and the calculated average spectra were used for further analysis. The interference of H_2_O and CO_2_ in the air should be deducted when the blank KBr slice was scanned. The collected FT-IR raw spectra data usually contain a lot of noise and interference information. In order to achieve good model performance, different preprocessing and feature variable extraction methods were needed to optimize the dataset. The combination of multiplicative scatter correction (MSC) and second derivative (SD) was selected to reduce the particle size and correct the baseline drift effect (Dhanoa et al., 2017; Zhan et al., 2017). SIMCA-P^+^14.1 software was used for performing all pretreatment steps.

### Chromatographic Determination

Quantitative analysis was carried out with an Agilent 1260 Infinity HPLC system (GL Sciences Company, Japan) that equipped with a G1311C quaternary pump, an Agilent Intersil-C18 column (150 × 4.6 mm, 5 µm) and the G1329B ALS automatic sampling device connected to a G1315D DAD VL detector. The gradient elution of eluent A (0.1% formic acid aqueous solution) and B (acetonitrile) was performed for the isolation of the target analytes. The flow rate of solvent was 1 ml·min^-1^ and the gradient elution procedure was as follows: 0–2.5 min, 93–90% A; 2.5–20 min, 90–74% A; 20–29.02 min, 74–41.7% A; 29.02–30 min, 41.7–10% A; and 30–34 min, 10–93% A. There was a four-min balancing time after each run. The column temperature was set at 30°C and the detected wavelength was chosen at 241 nm. Each extraction was analyzed with the same volume of 5 µl.

### Preparation of the Sample Solutions and Standard Solutions

Each sample powder (0.0250 ± 0.0005 g) of root tissues was weighted accurately through AR1140 Ohaus electronic analytical balance (Shanghai, China). Then it was put into a cuvette with stopper and added into 1.5 ml of 80% methanol, treating with the SY3200-T ultrasonic bath (40 kHz) for 40 min (Shanghai Shengyuan Instrument Equipment, Shanghai, China). The extract solution was filtered by the 0.22 µm micro-porous membrane filter before injecting into the HPLC system for analysis.

For the calibration standard preparation of five characteristic compounds (LA, GE, GG, ST, and SW), the standard solution was accurately extracted 1 ml and appropriately added with chromatographic methanol to dilute it into a series of consecutive concentrations (0.0044–0.8001, 0.0040–0.7998, 0.0018–0.7683, 0.0498–3.6000, 0.0018–0.7001 mg·ml^-1^, respectively). The levels of signal-to-noise ratio (SNR) are set equal to three and ten represented the parameters of limit of detection (LOD) and limit of quantification (LOQ) respectively, which were measured by taking an appropriate amount of mix standard solution and diluting with chromatography methanol to a serial of concentrations mixture. All prepared solutions were stored in a refrigerator at 4°C and reached room temperature before analysis.

### Methodological Validation of Quantitative Analysis

The calibration standard curve, LOD, and LOQ were experimentally verified for each standard composition of this method. A mixture solution of five analytes was diluted to the appropriate concentrations for the construction of calibration curves. The repeatability, stability, and precision were experimentally validated by analyzing the mixed standard solutions. As the evaluation of precision in intra- and inter-day, the mixed standard solution was analyzed six times within a day and 3 consecutive days. The stability was determined by analyzing the mixed standard solutions six times (0, 4, 8, 12, 16, 20, and 24 h) during a day. For the repeatability, six independent mixed standard solutions were analyzed by repeating the described procedure of mixed standard solutions preparation under this chromatographic condition. The accuracy of chromatographic analysis method was validated by the recovery test. Three different amounts (low, medium, and high) of each standard solution were added into the extraction of sample Nanhua Chuxiong (36). The recovery rates were calculated as follows:

Recovery (%)=(measured amount-original amount )/amount added ×100%

### Collection of Environmental Factors

The geographical information including longitude, latitude and altitude were recorded by GPS (HC 608) during collecting samples. All of the climate data were obtained from “China Meteorological Data Network” (http://data.cma.cn/site/index.html), including average annual temperature, 5–7 monthly mean temperature, average annual precipitation, and 5–10 monthly mean precipitation. These environmental factors were chosen according to the growth habit of *G. rigescens* (Chen, 2001). The information related to environmental factors and chemical data were dealt with by Excel (2013) software and transformed by log10(x) to eliminate the interference of dimension difference. As shown in **Figure 1**, the geographical distribution of samples was realized by ArcGIS 10 (ESRI Inc., USA).

### Multisource Data Analysis

In this study, based on the classical Kernnard-Stone algorithm (Kennard and Stone, 1969), 158 samples from four prefecture-level cities in Yunnan Province were divided into two-thirds training sets (118 individuals) and one-third test sets (40 individuals). The training sets were used to construct the classification model, and the test sets were used to estimate the classification performance of the model. PLS-DA and SVM algorithms were selected to establish calibration models for 158*1867 original spectral variables under seven-fold cross-validation. At the same time, the effects of preprocessing, feature extraction and their combination of spectral information mining were compared. PLS-DA is a supervised linear classification method based on the theory of the PLS regression algorithm, which contributes to the geographical tracking effectively of *G. rigescens*. The parameters R^2^Y(cum) and Q^2^(cum) (the cumulative R^2^ and Q^2^ for the extracted components) are used to predict the degree of data matching with the model. In general, it is considered that the model with Q^2^(cum) greater than 0.5 has better prediction ability. According to the root mean square error of cross-validation (RMSECV) and root mean square error of prediction (RMSEP), the stability of the model is evaluated. The condition for model fitting required RMSECV to be greater than RMSEP and the model had better stability and prediction ability if the values were smaller (Berrueta et al., 2007). In addition, SVM can achieve better classification results for some complex nonlinear classification problems (Chen et al., 2007; Martelo-Vidal et al., 2013). Then, penalty parameter coast (c) and kernel parameter gamma (g) are important parameters that affect SVM classification performance. Grid search (GS) and Genetic algorithm (GA) are the methods for finding the optimal combination of parameters, which can quickly find the optimal solution in complex parameter space. PLS-DA and SVM models were carried out for geographical traceability of wild *G.rigescens* by SIMCA-P^+^ (Version 14.1, Umetrics, SWE) and MATLAB (Version 2017A, MathWorks Inc, USA) software.

In addition, PCA, the unsupervised learning algorithm, can reveal the correlation among analytical parameters and visualize the classification trend of samples (Pei et al., 2019). HCA also can be regarded as the common method to uncover the similarities among samples, which calculates the dissimilarity between two patterns through measurement of distance. The nearest data or categories are combined to generate the cluster tree and the smallest distance has the highest similarity. ANOVA is a statistical method used to compare the mean of dependent variables in different groups. CA can be used to find the correlation between two variables. According to the visualization results of the above-mentioned algorithms, the effects of different environmental factors on the chemical components of *G. rigescens* can be preliminarily explored. In this study, the variable factor plot of PCA was performed by the package of “FactoMineR,” which was used to reveal the relationship between environmental factors and the variable of five iridoids. HCA was established to explore the difference and similarities of samples by the package of “Dendextend.” The interaction among chemical components extracted from various region samples was investigated by the multiple trials of ANOVA, which was carried out by the package of “Multcomp.” CA was achieved by the package of “Hmisc” so as to study the effect of environmental factors on the contents of five iridoids. The analysis of ANOVA, CA, PCA, and HCA were performed by RStudio 3.4 software.

## Results

### Interpretation of FT-IR Spectra Features

**Figure 3:**
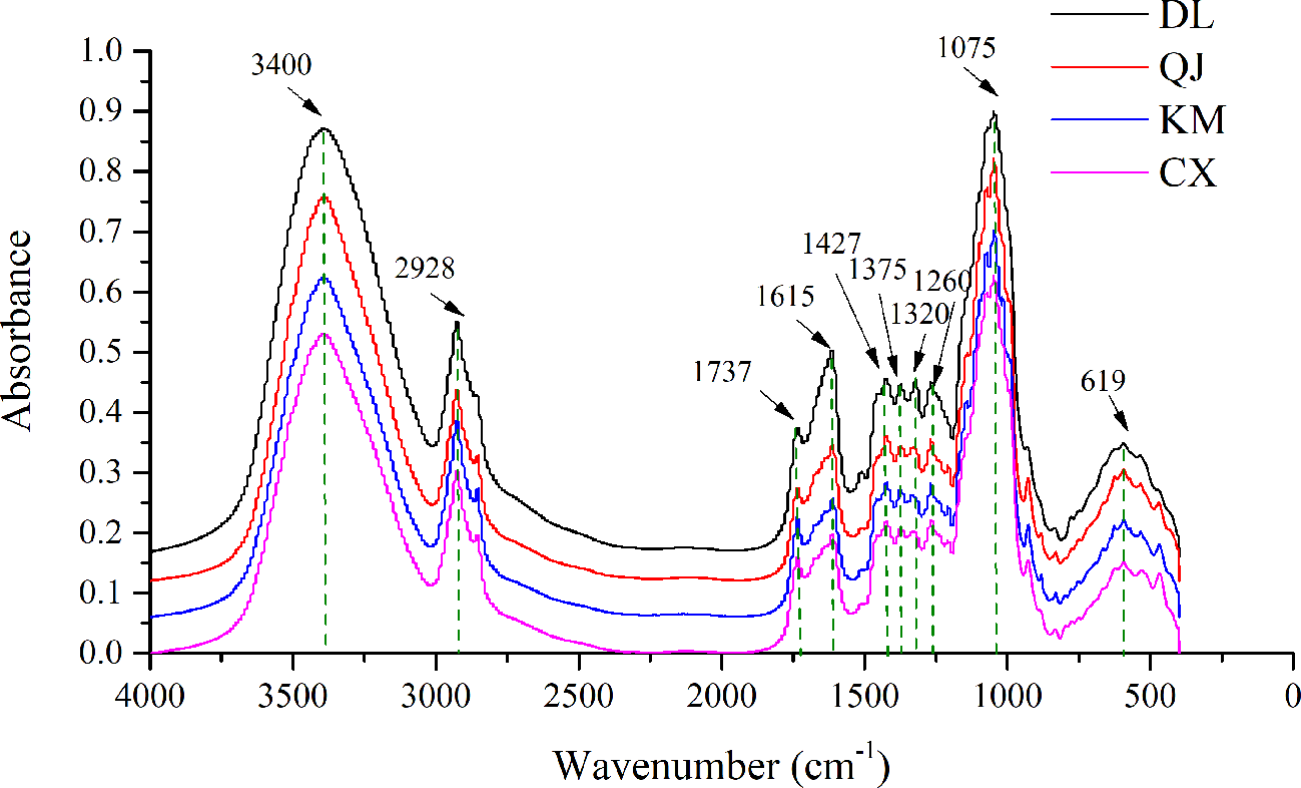
Fourier transform infrared (FT-IR) spectra of *Gentiana rigescens* roots from four geographical regions. DL, Dali; QJ, Qujing; KM, Kunming; CX, Chuxiong.

The averaged FT-IR spectra of 158 *G. rigescens* samples from four different geographical origins were shown in **Figure 3**. Obviously, the overlapped average FT-IR spectra have some similar characteristic peaks, but there are differences in absorption intensity which implies differences in the accumulation of chemical components from different regions. Compared with other geographical origins, the absorption intensity increased significantly in the root samples of Dali. The obvious peak at 3,400 cm^-1^ was assigned to the first overtone with O-H stretching. The bands around 2,928 and 1,737 cm^-1^ represented methylene asymmetric and C = O stretching vibration of esters respectively, which were mainly caused by esters. The band at 1,427 cm^-1^ represented the asymmetric bending vibration of methyl group. This was the result of carbohydrates or esters. The peak at 1,375 cm^-1^ showed that the bending vibration of methyl group was mainly caused by esters. In addition, the sharpest peaks at 1,075 and 1,615 cm^-1^ were the main absorption bands of GE, belonging to C-OH or C-O stretching and C-C asymmetric stretching vibration. Anyway, since the metabolic components of *G. rigescens* are very similar, multivariate analysis methods were needed for the next analysis (Mi et al., 2015; Zhao et al., 2015).

### PLS-DA and SVM Analysis for Geographical Origin Identification

In order to develop a reliable discriminant model, some samples were used to build the model and others were used for validation. It was closely related to the robustness of the model (Ballabio and Consonni, 2013). The results that RMSECV values of all PLS-DA models were greater than RMSEP indicated the reliable model.

To validate the fitting degree of PLS-DA models, the 20-iteration permutation test were performed. As shown in **Figure S1** that all permutated Q^2^ and R^2^ values (left) were lower than the original values (right). It showed that all models were regarded to be suitable without overfitting. However, all Q^2^ values less than 0.5 indicated the weak prediction ability. The classification performance of PLS-DA model with preprocessed spectra was better than the original spectral model. The accuracy of training and validation set was 93.22 and 95%, respectively. However, the classification effect among different categories was not ideal in the scatter plot. Despite the combination of preprocessing and feature extraction method, there was still a big confusion between QJ and KM. PLS-DA model was established by selecting the variables with variable importance for the projection (VIP) score greater than 1 for improving classification effect. However, the accuracy of the correction set and the verification set after feature filtering was less than 70%, which showed that this variable filtering method was not suitable for this data. The separation results of calibration set and validation set of all models were shown in **Table 2** and **Figure S2**.

**Table 2:** Classification results of partial least squares discriminant analysis (PLS-DA) model using Fourier transform infrared (FT-IR) spectra.

Model types	R^2^Y(cum)	Q^2^(cum)	RMSEE	RMSECV	RMSEP	Accuracy of training set (%)	Accuracy of test set (%)
Raw	0.4441	0.2943	0.2563	0.3099	0.2406	72.88	77.50
Raw+ VIP	0.3933	0.2696	0.2476	0.2938	0.2276	68.64	70.00
MSC+2D	0.7003	0.4407	0.2432	0.2701	0.1215	93.22	95.00
MSC+2D + VIP	0.3700	0.3088	0.2579	0.2660	0.2390	66.95	67.50

**Figure 4:**
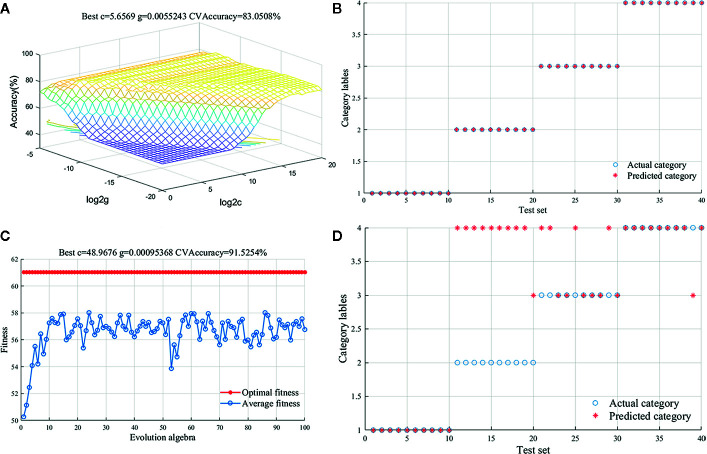
Support vector machine (SVM) results for preprocessed spectral data. **(A)**: Parameter selection diagram with the method of Grid search (GS); **(B)**: Classification result diagram of test set with the method of GS; **(C)**: Fitness curve with the method of Genetic Algorithm (GA); **(D)**: Classification result diagram of test set with the method of GA.

Compared to the method of PLS-DA, SVM had the better performance to deal with nonlinear problems. Thus, SVM was also used to discriminate *G. rigescens* samples from different producing areas based on FT-IR spectra data in this study. The assign of training and test samples was same as the PLS-DA model. In addition, Radial Basis Function (RBF) was preferred to build SVM model for handling the nonlinear and linear relationships between spectra data (Zhao et al., 2009). To attain a good classification result, penalty parameter c and kernel parameter g were used to optimize the SVM model. The best c and g of 5.6569 and 0.0055 were confirmed by the GS method in the preprocessed model (**Figure 4A**). The 83.05% accuracy of training sets and 100% accuracy of test set were acquired in calibration model (**Figure 4B**). It meant that 20 samples were misclassified in the training set and all 40 unknown samples were classified correctly in the test set. **Tables S4 and S5** provided the confusion data matrix of training set and test set of PLS-DA and SVM models. Compared with the model built directly from the original data, the classification performance was significantly improved. In addition, other parameter optimization methods have been tried to distinguish *G. rigescens* from different origins in SVM. **Figure 4C** showed that when the genetic algorithm run to the 10th generation, the average fitness value tended to be stable, while the optimal fitness value kept constant with the increase of iteration times. However, based on the method of GA, the accuracy of test sets was only 52.5 and 62.5% in the original and pre-processed model (**Figure 4D**). The classification results of SVM models with different processing methods were shown in **Table 3**. In conclusion, compared with PLS-DA method, the SVM-GS can show more superior performance for the discrimination of *G. rigescens* from different producing areas. As well, the selections of spectra pretreatments and some parameters are important for a well calibrated SVM model.

**Table 3:** Classification results of support vector machine (SVM) model using Fourier transform infrared (FT-IR) spectra.

Model types	Best c	Best g	The accuracy of training test (%)	The accuracy of test set (%)
Raw-GS	0.7071	0.0313	61.02	75.00
Raw-GA	0.7274	1.3251	61.86	52.50
MSC+2D-GS	5.6569	0.0055	83.05	100.00
MSC+2D-GA	48.9676	0.0010	91.53	62.50

### Quantitative Analysis of Five Iridoids

The external standard method was carried out to quantify the contents of five iridoids. All standard solutions were diluted by methanol to an appropriate concentration for establishing the regression equation, which were plotted with seven different concentrations and revealed good linear relationship (R^2^ > 0.9991) (**Table S1**). The relative standard deviations (RSDs) of precision, stability, and repeatability were reasonable and desirable (RSD < 5%), indicating that the analytical instrument, extraction, and analysis method used in this experiment are reproducible (**Table S2**). Additionally, the results of recovery test for analyzing the five iridoids were in the range of 97.2–100.1% and the RSD values were inferior to 1.18%, which indicated that this method was creditable for quantitative analysis (**Table S3**).

**Figure 5:**
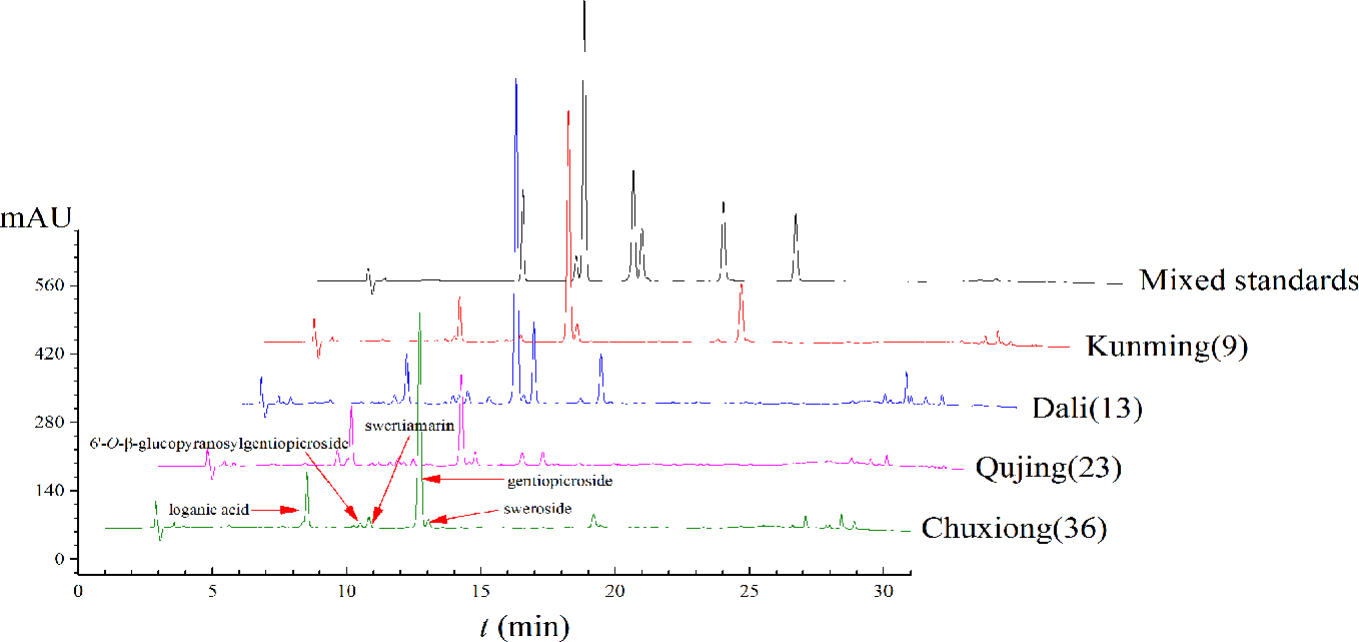
The raw chromatograms of *Gentiana rigescens*.

In this study, LA, ST, GE, SW, and GG were the main compounds detected in the root tissues of *G. rigescens*. However, the Chinese Pharmacopeia merely takes the contents of GE as the threshold to evaluate the quality of *G. rigescens* (National Pharmacopoeia Committee, 2015), which presents with some insufficiency due to the holistic characteristic of TCM. Therefore, finding out the evaluation indicator as many as possible was meaningful for establishing a comprehensive method to evaluate the quality of medicinal materials. In this study, the chromatographic peak of sample obtained from Nanhua Chuxiong (36), Luoping Qujing (23), Eryuan Dali (13), and Xundian Kunming (9) were measured and five iridoids were chosen as the target analytes to evaluate the quality of *G. rigescens* by comparing and validating the retention times of reference standards (**Figure 5**).

**Figure 6:**
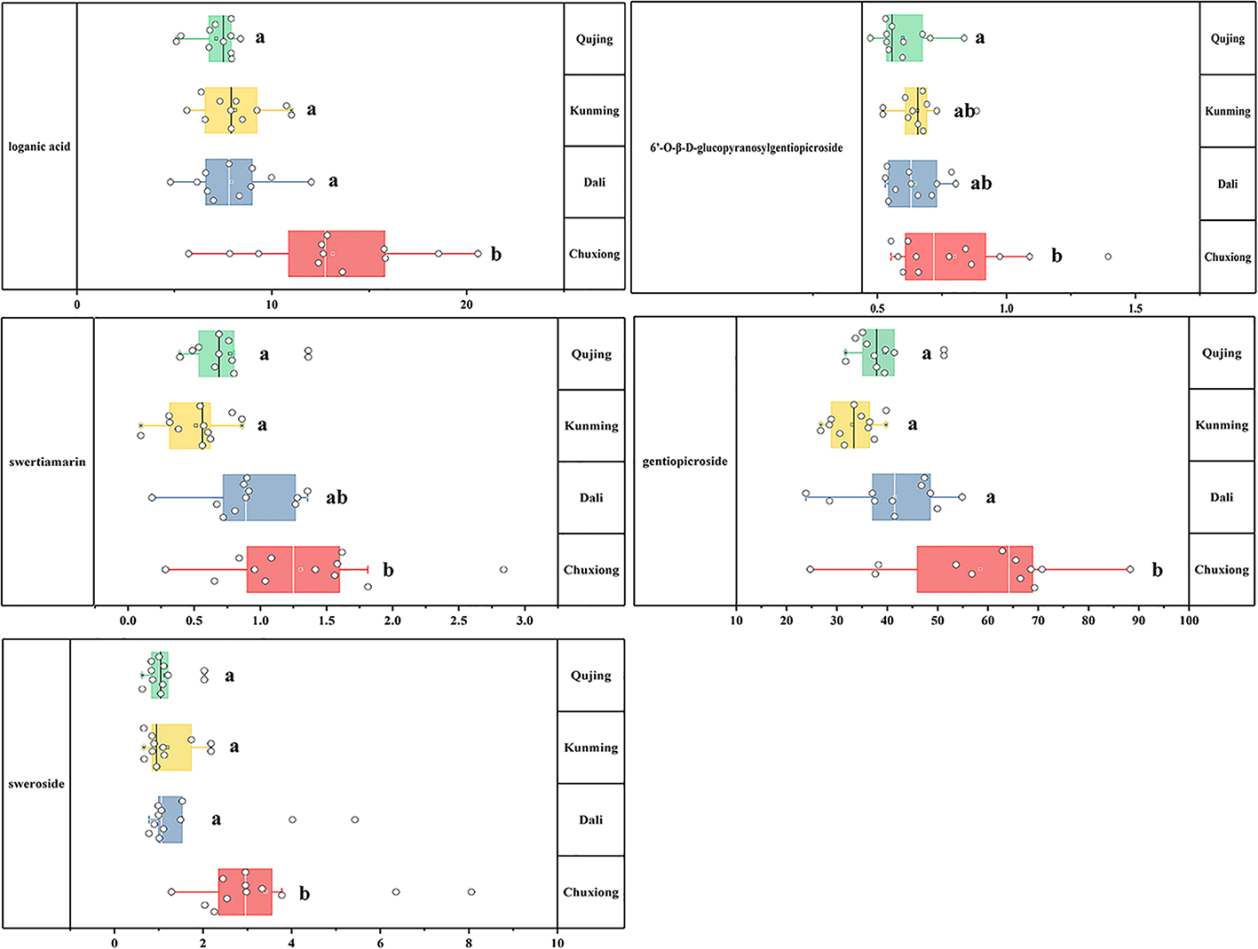
The result of One-way analysis of variance (ANOVA) for iridoid content. The significance differences level was 0.05. LA, loganic acid; GG, 6'-*O*-β-D-glucopyranosylgentiopicroside; ST, swertiamarin; GE, gentiopicroside; SW, sweroside. Different letters show significant difference (p < 0.05).

**Figure 7:**
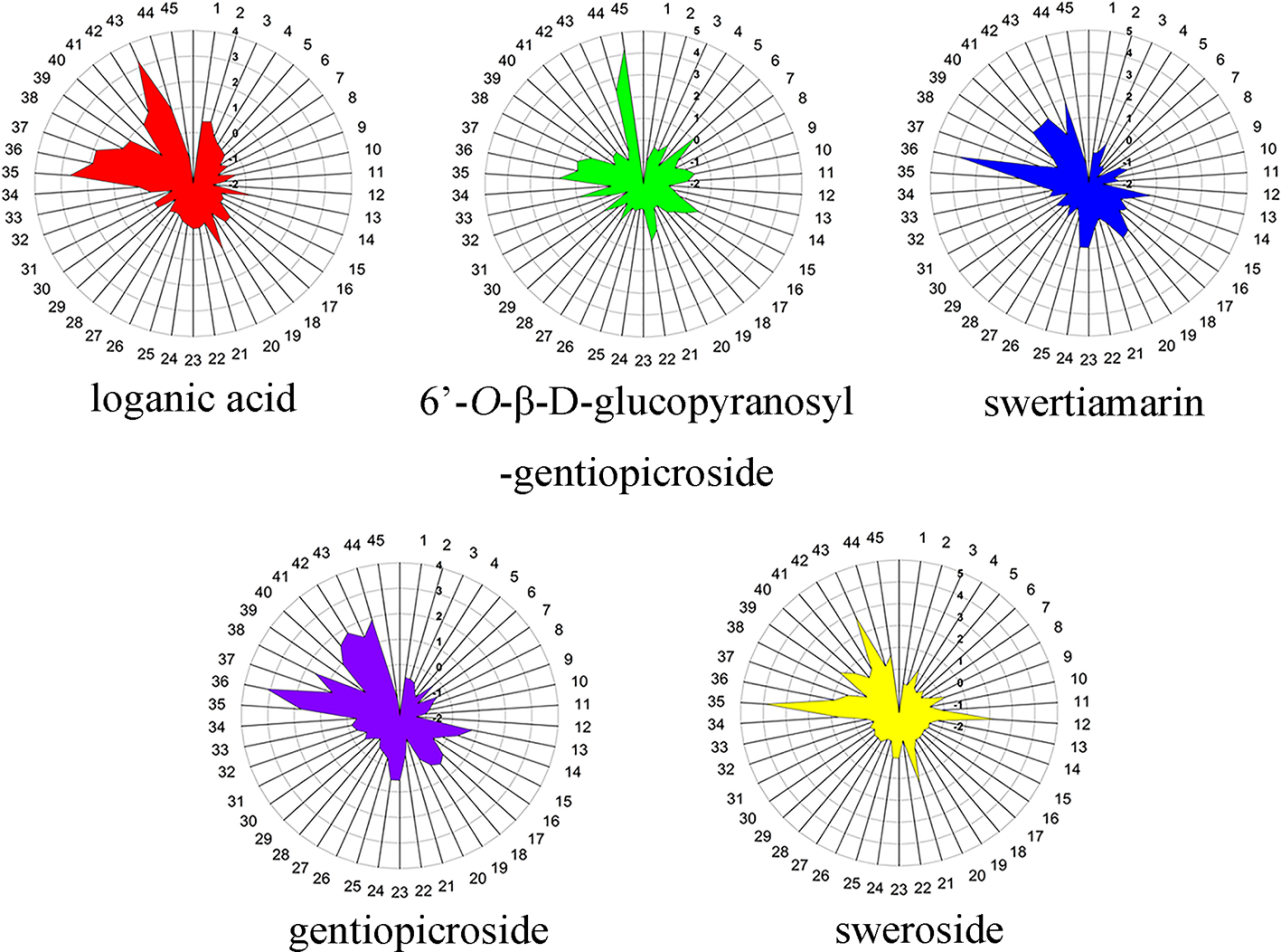
The radar map for five iridoid constituent content in root tissue of *Gentiana rigescens*. The number of 1–45 represent the corresponding locations.

One-way analysis of variance was computed to display the difference of iridoids among four origins (**Figure 6**). Compared with other four chemical components, the contents of ST exhibited the most fluctuating characters (**Figure 7**). The highest content (2.84 mg·g^-1^) of ST was detected in sample of Nanhua, Chuxiong (36), which was approximately 29 times than the lowest one (0.10 mg·g^-1^) measured in the sample of Xundian Kunming (7) (**Table 4**). For the GG, the highest content (1.39 mg·g^-1^) was measured in the sample of Lufeng Chuxiong (45), which was merely three times than that of the lowest one (0.47 mg·g^-1^) collected from Fuyuan, Qujing (31) (**Table 4**). The ratios of the highest content to the lowest content for other three constitutes of SW, LA, and gentiopcroside are 13.0, 4.3, and 3.7 times, respectively (**Table 4**). The above-discussed results indicated that the contents of five iridoids measured in the root tissue of *G. rigescens* was remarkably varied among different origins. Additionally, the GE content ranged from 24.84 to 88.27 mg·g^-1^, which was significantly higher than the threshold value of the Chinese Pharmacopoeia (the contents of GE > 1.5 mg·g^-1^). Interestingly, the highest content of these five iridoids was exhibited in the sample obtained from Chuxiong (**Figures 5** and **7** and **Table 4**). It can be seen that different origins have great influence on the accumulation of five iridoids in the root tissue of *G. rigescens*.

**Table 4:** Mean content ± standard error (mg·g^-1^) of five iridoids in root tissue of *Gentiana rigescens*.

Number	Loganic acid	6'-*O*-β-D-glucopyranosylgentiopicroside	Swertiamarin	Gentiopicroside	Sweroside
1	10.75 ± 1.27	0.52 ± 0.04	0.57 ± 0.05	36.49 ± 1.83	0.89 ± 0.08
2	11.01 ± 1.55	0.64 ± 0.05	0.60 ± 0.10	36.20 ± 1.86	0.86 ± 0.12
3	9.23 ± 1.40	0.62 ± 0.07	0.86 ± 0.13	34.82 ± 1.72	2.18 ± 0.51
4	8.49 ± 0.76	0.69 ± 0.04	0.32 ± 0.04	30.57 ± 1.39	0.85 ± 0.09
5	8.17 ± 1.01	0.52 ± 0.02	0.38 ± 0.11	28.50 ± 1.87	0.67 ± 0.14
6	7.89 ± 0.73	0.88 ± 0.06	0.31 ± 0.04	39.78 ± 1.66	1.09 ± 0.09
7	6.58 ± 1.23	0.66 ± 0.06	0.10 ± 0.04	26.78 ± 2.09	0.66 ± 0.09
8	7.33 ± 1.09	0.61 ± 0.05	0.62 ± 0.08	37.41 ± 1.19	1.12 ± 0.22
9	5.63 ± 0.64	0.68 ± 0.07	0.79 ± 0.12	33.39 ± 2.08	2.18 ± 0.39
10	7.92 ± 0.76	0.73 ± 0.10	0.44 ± 0.16	31.47 ± 2.91	0.95 ± 0.09
11	6.37 ± 0.86	0.68 ± 0.05	0.54 ± 0.08	28.87 ± 1.89	1.73 ± 0.22
12	4.79 ± 0.35	0.53 ± 0.06	0.18 ± 0.06	23.84 ± 2.28	5.43 ± 4.05
13	9.98 ± 0.74	0.57 ± 0.04	1.28 ± 0.08	54.88 ± 2.68	1.49 ± 0.26
14	6.17 ± 0.54	0.63 ± 0.06	0.89 ± 0.08	48.56 ± 1.99	1.00 ± 0.14
15	6.69 ± 1.12	0.80 ± 0.11	0.67 ± 0.11	41.02 ± 2.37	1.11 ± 0.12
16	6.60 ± 0.96	0.73 ± 0.07	0.91 ± 0.08	37.03 ± 1.26	0.90 ± 0.07
17	8.92 ± 0.72	0.66 ± 0.02	0.81 ± 0.08	46.81 ± 2.01	0.99 ± 0.10
18	8.99 ± 1.24	0.62 ± 0.07	1.27 ± 0.14	49.95 ± 3.47	1.06 ± 0.13
19	7.00 ± 0.74	0.54 ± 0.05	1.36 ± 0.16	47.38 ± 2.43	1.01 ± 0.12
20	12.02 ± 1.19	0.54 ± 0.05	0.87 ± 0.11	41.45 ± 3.28	1.53 ± 0.22
21	7.80 ± 0.54	0.71 ± 0.05	0.72 ± 0.08	28.53 ± 1.69	4.02 ± 0.66
22	8.32 ± 1.50	0.79 ± 0.06	0.90 ± 0.06	37.50 ± 1.87	0.78 ± 0.13
23	8.39 ± 1.60	0.54 ± 0.05	1.36 ± 0.13	51.25 ± 2.20	2.03 ± 0.97
24	7.90 ± 1.12	0.54 ± 0.03	1.36 ± 0.11	51.25 ± 2.66	2.03 ± 0.10
25	7.51 ± 1.24	0.56 ± 0.04	0.69 ± 0.11	39.64 ± 2.24	0.83 ± 0.16
26	6.81 ± 1.05	0.54 ± 0.04	0.49 ± 0.02	37.39 ± 2.18	0.86 ± 0.06
27	6.77 ± 1.16	0.67 ± 0.08	0.79 ± 0.13	35.92 ± 2.88	1.28 ± 0.17
28	7.10 ± 0.71	0.53 ± 0.03	0.53 ± 0.07	31.73 ± 0.87	1.09 ± 0.11
29	5.33 ± 0.85	0.60 ± 0.04	0.66 ± 0.12	33.68 ± 2.06	1.11 ± 0.18
30	7.91 ± 1.46	0.70 ± 0.03	0.76 ± 0.11	37.86 ± 1.68	0.83 ± 0.09
31	7.92 ± 1.25	0.47 ± 0.03	0.39 ± 0.06	35.10 ± 1.88	1.04 ± 0.17
32	5.10 ± 1.03	0.60 ± 0.07	0.80 ± 0.15	39.46 ± 2.64	0.63 ± 0.08
33	7.94 ± 0.87	0.84 ± 0.07	0.69 ± 0.08	41.40 ± 1.60	1.00 ± 0.14
34	9.32 ± 1.18	0.58 ± 0.02	0.96 ± 0.14	38.24 ± 2.13	2.98 ± 0.26
35	18.56 ± 1.46	0.97 ± 0.02	1.42 ± 0.04	68.52 ± 1.51	8.06 ± 0.68
36	15.77 ± 0.92	0.84 ± 0.04	2.84 ± 0.08	88.27 ± 1.55	3.33 ± 0.29
37	15.82 ± 1.40	0.86 ± 0.04	1.04 ± 0.10	53.67 ± 2.68	2.54 ± 0.32
38	12.64 ± 1.05	0.78 ± 0.07	0.65 ± 0.05	66.46 ± 1.41	1.29 ± 0.04
39	12.39 ± 2.07	0.60 ± 0.05	0.84 ± 0.15	37.63 ± 2.83	3.78 ± 1.10
40	7.84 ± 0.98	0.65 ± 0.02	1.58 ± 0.09	56.80 ± 2.26	2.96 ± 0.23
41	12.56 ± 0.92	0.66 ± 0.04	1.56 ± 0.08	65.55 ± 1.95	2.04 ± 0.09
42	13.62 ± 0.95	0.55 ± 0.03	1.62 ± 0.08	69.26 ± 2.29	2.45 ± 0.13
43	20.58 ± 0.85	0.62 ± 0.02	1.08 ± 0.08	62.88 ± 2.14	6.35 ± 0.32
44	12.84 ± 0.58	1.08 ± 0.05	1.81 ± 0.05	70.74 ± 1.32	2.25 ± 0.27
45	5.72 ± 1.52	1.39 ± 0.06	0.31 ± 0.11	24.69 ± 2.24	2.96 ± 0.11

**Figure 8:**
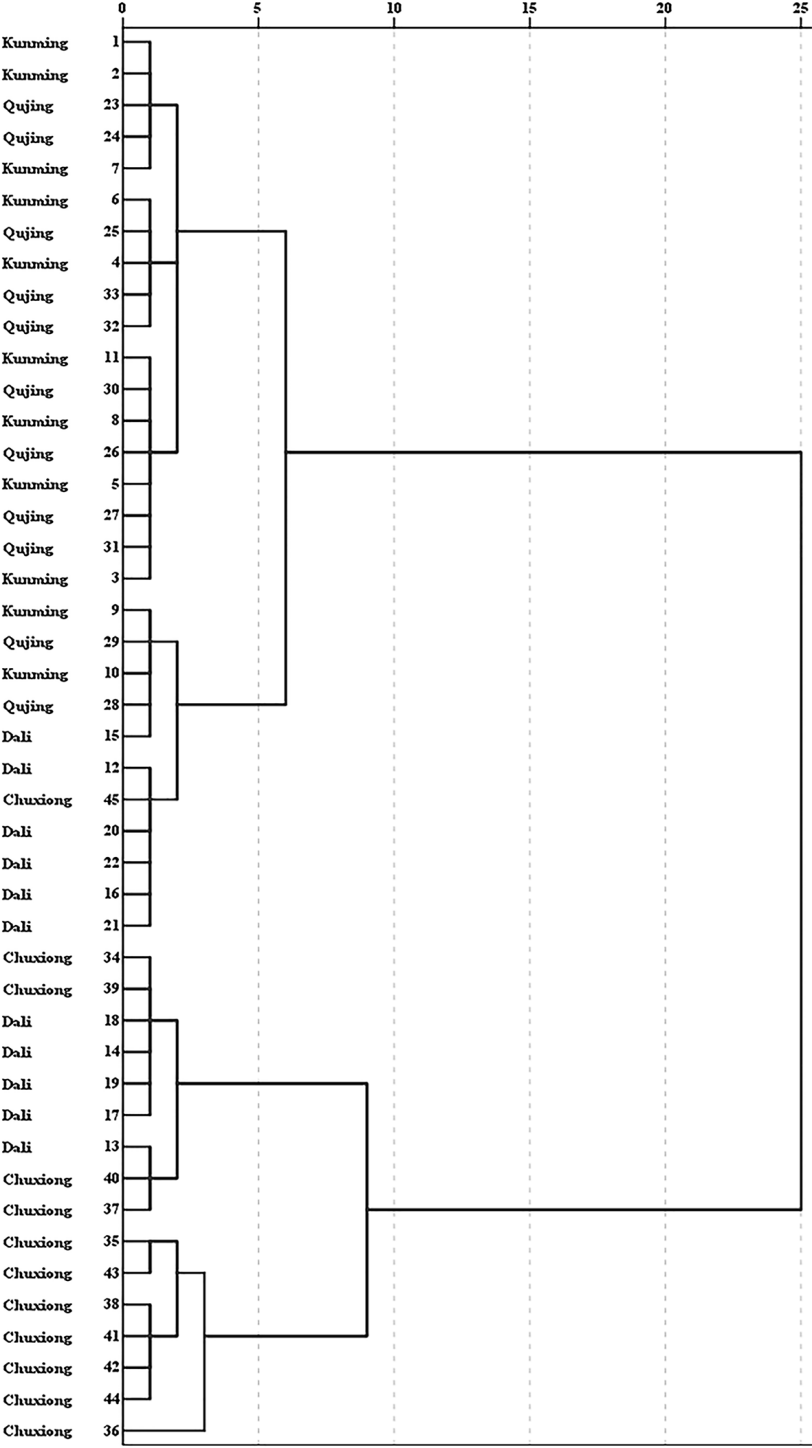
Cluster analysis of *Gentiana rigescens* from different origins. The number of 1–45 represent the corresponding locations.

HCA was performed to expose the similarities of *G. rigescens* obtained from different origins based on the five iridoid content (**Figure 8**). The result showed that 45 batch wild samples had a tendency to be divided into two categories. Almost all of the samples obtained from Chuxiong were clustered into one class, which were close to the *G. rigescens* samples collected from Dali, suggesting that the chemical profiles of *G. rigescens* between these two origins are similar. However, samples obtained from Kunming and Qujing were disorganized assembled, indicating that the similarity of iridoid content in these two origins was higher than that of samples picked from Chuxiong and Dali. This unsatisfactory classification result for *G. rigescens* obtained from Kunming and Qujing indicated that the difference of iridoid content between these two prefectures regions was insignificant, coinciding with the result of ANOVA.

### Correlation Between Environmental Factors and Five Iridoids

To explore the relationship between environmental factors and the iridoid content, correlation analysis was carried out and the results were shown in **Table 5**. The LA was significantly affected by the environmental factors of altitude (*P* < 0.01) and 5–7 monthly mean temperature (*P* < 0.05). Besides, iridoids of ST, GE, and GG were influenced by longitude (*P* < 0.05). Interestingly, component of SW was remarkably affected by almost all the factors including altitude, average annual temperature, 5–7 monthly mean temperature and 5–10 monthly mean precipitation (*P* < 0.01). However, the altitude environmental factor was negative to the accumulation of these five iridoids, particularly for components of LA and SW (*P* < 0.01). Altitude showed a significantly negative relationship with the average annual temperature and 5–7 monthly mean temperature (*P* < 0.01), which was in correspondence with common sense. The information also could be summarized that the average annual temperature and 5–7 monthly mean temperature could promote the accumulation of these five iridoids, especially for SW (*P* < 0.01). With the increase of precipitation, the contents of these five iridoids decreased, particularly for SW (*P* < 0.05). Besides, an inner-correlation among different iridoids measured in root tissue of *G. rigescens* was revealed and results were shown in **Table 5**. The LA was significantly positive correlation with ST and GE (*P* < 0.01). The SW was positive to LA (*P* < 0.05). ST showed a significantly positive correlation with SW and GE (*P* < 0.01). GE had a positive correlation with SW (*P* < 0.05). Findings indicated that the accumulation of these five iridoids might be closely related.

**Table 5:** Correlation analysis between environmental factors and five iridoids in root tissue of *Gentiana rigescens*.

Factors	A	B	C	D	E	F	G	LA	GG	ST	GE	SW
A	1.000	−0.464^**^	0.412^**^	−0.187	−0.201	−0.458^**^	0.054	−0.343^*^	−0.081	0.096	−0.019	0.023
B		1.000	−0.325^*^	0.393^**^	0.257	0.357^*^	−0.186	0.160	0.329^*^	−0.331^*^	−0.320^*^	0.018
C			1.000	−0.456^**^	−0.535^**^	−0.133	0.490^**^	−0.453^**^	−0.077	−0.202	−0.221	−0.489^**^
D				1.000	0.923^**^	0.210	−0.556^**^	0.198	0.267	0.004	0.014	0.512^**^
E					1.000	0.055	−0.573^**^	0.308^*^	0.221	0.164	0.136	0.651^**^
F						1.000	0.346^*^	−0.049	−0.090	−0.224	−0.257	−0.324^*^
G							1.000	−0.257	−0.257	−0.194	−0.154	−0.649^**^
LA								1.000	0.104	0.499^**^	0.614^**^	0.321^*^
GG									1.000	0.078	0.151	0.146
ST										1.000	0.811^**^	0.436^**^
GE											1.000	0.310^*^
SW												1.000

The abbreviations are follow as: A, latitude; B, longitude; C altitude; D, average annual temperature; E, 5–7 monthly mean temperature; F, average annual precipitation; G, 5–10 monthly mean precipitation; LA, loganic acid; GG, 6'-O-β-D-glucopyranosylgentiopicroside; ST, swertiamarin; GE, gentiopicroside; and SW, sweroside, respectively. Significant differences (P < 0.05) marked as “*”, (P < 0.01) marked as “**”.

**Figure 9:**
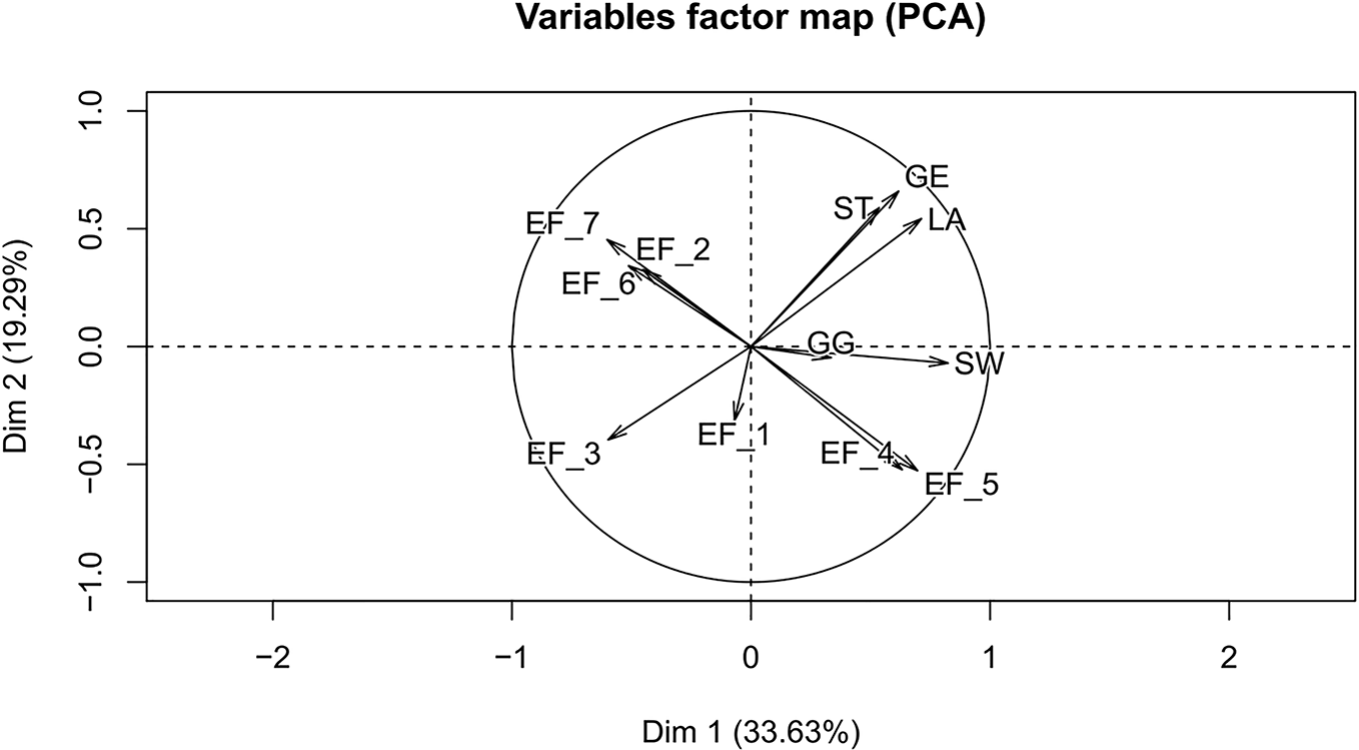
The variables factor map between environmental factors and iridoids. EF_1, latitude; EF_2, longitude; EF_3, altitude; EF_4, average annual temperature; EF_5, 5–7 monthly mean temperature; EF_6, average annual precipitation; EF_7, 5–10 monthly mean precipitation; LA, loganic acid; GG, 6'-*O*-β-D-glucopyranosylgentiopicroside; ST, swertiamarin; GE, gentiopicroside; SW, sweroside.

In the present study, PCA was used to reveal the relationship between environmental factors and the five iridoids, which could verify and testify the result of correlation analysis. The variables factors map of PCA was shown in **Figure 9**. Obviously, the average annual temperature and 5–7 monthly annual temperature were positive for the accumulation of these five iridoids, especially for SW and GG. However, the altitude, average annual precipitation, and 5–10 monthly annual precipitation were adverse for the storage of these five constituents for the root tissue of *G. rigescens,* remarkably for the components of LA, ST, and GE. The results of variables factors map for PCA were consistent with the correlation analysis, indicating that the results of statistical analysis were reliable.

## Discussion

### The Variety of Iridoid Content in Root Tissue of *G. rigescens* From Different Origins

The species, quantity, and quality of TCM across different producing areas can be quite different. Typically, the intraspecific variation among populations is the fundamental factor affecting the yield and quality of TCM materials (Bowling et al., 2002). In this study, multivariate analysis was carried out to mine the FT-IR spectral information, which was regarded as an effective approach to authenticate the geographical origin of gentian samples. Compared with the PLS-DA model, SVM showed better classification performance, which might be due to the fact that it maximally eliminated the influence of interference information. In addition, based on the quantitative analysis of *G. rigescens* samples collected from four main origins of Yunnan, different origins greatly affected the accumulation of five iridoids in the root tissue of *G. rigescens*. Additionally, all these five iridoids with the highest contents were obtained from Chuxiong consistently, indicating that Chuxiong might be one of the best cultivation areas for this Chinese herbal medicine. Meanwhile, according to the analysis of habitat, it was speculated that the high content of iridoids might be induced by the temperature in Chuxiong, where the day/night temperature differences were greatly different, especially for the long-day spring and autumn seasons. Under such condition, more secondary metabolites were accumulated during the daytime, but less were consumed at night. Besides, the precipitation of Chuxiong was lower than that of the other four origins in Yunnan (Akula and Ravishankar, 2011), which was beneficial for producing metabolites, and such hypothesis was confirmed by other studies (Bowling et al., 2002). However, the similarities of samples collected from various locations were insignificant, even under the same climatic zone. Interestingly, the chemical profiles for samples obtained from Chuxiong and Dali showed similar characteristics, which might be attributed to the similar geographical environment in these two origins. **Table 1** displays the numerical similarities of annual average temperature in Chuxiong and Dali, and all these values are higher than those in Kunming and Qujing. Compared with the environmental factor of temperature, the annual average precipitation values in Chuxiong and Dali were lower than those in Kunming and Qujing. Significantly, the origin of Chuxiong represented a favorite environment for accumulating the secondary metabolites in the root tissue of *G. rigescens*, which was also meaningful for the medicinal materials with better quality. Based on the above-mentioned analysis results, it was meaningful to effectively identify the geographical origin to create a cultivation environment similar to Chuxiong. Additionally, it was also significant to build a cultivation base in Chuxiong to satisfy the market demand of *G. rigescens* and to ensure the quality and efficiency of this medicinal material.

### Correlation Between Environmental Factors and Quality of *G. rigescens*

According to the correlations of seven environmental factors with the quality of *G. rigescens*, some environmental factors greatly affected the quality of *G. rigescens*, and there were significant correlations between these factors. In addition, the results of CA and PCA indicated that the average annual temperature and 5–7 monthly mean temperature were profitable for the accumulation of five iridoids, especially for SW. The underlying mechanism might be that the increase in temperature promoted the growth of meristems and organs, thus further accelerating the growth and development of plants (Morison and Lawlor, 1999). Another reason might be that the optimum temperature for metabolizing enzyme was achieved with the increase in ambient temperature (Treimer and Zenk, 1979). As a proof, Krithika et al. revealed that the optimum temperature for 10-hydroxygeraniol dehydrogenase (Cr10HGO) was 30°C, and its enzyme was a critical factor for synthesizing the intermediates in the biosynthetic pathway of iridoid (Krithika et al., 2015). Hence, within an appropriate range, high temperature might benefit the accumulation of iridoid substances in the root tissue of *G. rigescens*. Unquestionably, the environmental factor of altitude was negatively correlated with the contents of five iridoids, especially for LA and SW. Such phenomenon was possibly illustrated by the CA result that, the altitude showed a remarkably negative correlation with temperature. In other words, a higher altitude resulted in a lower temperature (Kattel et al., 2013). Chen et al. had drawn the same conclusion that the catechin content in oolong tea was negatively correlated with altitude, the content difference induced by temperature was low at a high latitude, and the enzymatic activity declined along with the increase in altitude (Chen et al., 2014). Overall, to achieve the higher quality and yield of *G. rigescens* raw material, the synergistic effects of environmental factors should be further investigated.

Precipitation is also a crucial factor that affects the quality of herbal medicine. In this study, the average annual precipitation and 5–10 monthly mean precipitation declined, which was beneficial for the accumulation of five iridoids, especially for SW. Jamieson et al. validated that, compared with *L. dalmatica* that grew in the condition of decreasing available water, the increase in soil available water decreased the average concentration of iridoid glycoside by about 35% (Jamieson et al., 2013). Other studies also find that the appropriately deficit irrigation induces the accumulation of secondary metabolites, such as lycopene and organic acids (Wang et al., 2015). Therefore, the cause of such phenomenon may be explained by the observation that, the appropriate drought stress is conducive to the accumulation of secondary metabolites (Chao and Ye, 2013). For instance, the camptothecin content is accumulated under drought stress, while the sustained drought stress decreased camptothecin content in leaf tissue (Liu, 2000). The above-mentioned phenomenon is consistent with the prediction of the growth-differentiation balance hypothesis, which uncovers that the synthesis and accumulation of secondary metabolites may increase under resource limiting conditions (Herms and Mattson, 1992). Based on the above discussion, theoretical results that, appropriate drought stress was meaningful for increasing the contents of secondary metabolites measured in the root tissue of *G. rigescens* during its cultivation process, was obtained. However, the precise soil moisture and air humidity should be further investigated. On the whole, findings in this study revealed that various environmental factors exhibited a synergistic effect on the growth of *G. rigescens*. Typically, the average annual temperature, 5–7 monthly mean temperature, the average annual precipitation and the 5–10 monthly mean precipitation played important roles in forming the *daodi* quality of *G. rigescens*.

Additionally, results of CA and PCA also uncovered the interrelationships among LA, ST, GE, and SW. Specifically, LA showed significantly positive correlation with the accumulation of ST, GE, and SW. This was because that, LA was the precursor of secoiridoid glycoside (Inouye, 1971; Liu et al., 2017). In other words, a higher content of LA was beneficial for the synthesis of its downstream products. ST also displayed significantly positive correlation with GE and SW. Besides, GE was positively correlated with SW. According to the analysis of structures, ST is the hydroxylated SW and GE can be formed by dehydration of ST (Inouye, 1971; Inouye et al., 1996). This revealed a general trend that, GE was the end metabolic product of these four iridoids, which was corresponding with the quantitative analysis result that the GE content was higher than those of LA, SW, and ST. Besides, Qi et al. confirmed that the root tissue of *G. rigescens* was the primary part for the accumulation of GE (Qi et al., 2017). Therefore, this quantitative analysis result accorded with the historically drug usage habit to select the root tissue for treating various ailments. On the other hand, high content of GE was accumulated, which might be the favorable defense substance in *G. rigescens* against environmental stress (Liu et al., 2017).

Our study clearly showed that there was a certain correlation of environmental factors with the quality of medicinal materials, and it might be a suitable method to find an alternative origin of medicinal materials according to the level of environmental factors. It was the reason that the change of environment in plant physiology could induce the physiological change of plants to produce the secondary metabolites (Su et al., 2005). Therefore, the difference of the temperature and precipitation existed between the planting land and the traditional land should be considered in the introduction, which can provide a theoretical basis for obtaining high-yield and high-quality resources. It is a complex process to explore the impact of environmental factors on the quality of traditional Chinese medicine, and single factor analysis is limited by its incompleteness. Thus, it is necessary to establish a systematic evaluation method including the role of a single factor and the interaction of various factors to deeply analyze the impact of environmental factors on the quality of medicinal materials, especially that in *daodi* origins. The analysis of all environmental factors of *daodi* medicinal materials is necessary to find the alternative or artificial habitats for herbs. It is our expectation that these findings can provide a basis for revealing the quality formation mechanism of *G. rigescens*, and provide a certain reference value for the collection, protection, development, and utilization of resources and selection and breeding of new varieties.

## Conclusion

Due to the non-destructive, fast, and efficient advantages of FT-IR spectra, multivariate analysis methods were used to identify *G. rigescens* samples from different areas in this study. SVM-GS was considered to be an effective method to trace the geographical origin. In addition, the relationship between five iridoid content in root tissue of *G. rigescens* and environmental factors under the same climatic zone was explored. Quantitative analysis direct uncovered that sample obtained from Chuxiong has better quality than the other three origins sample according to the highest content of iridoids in root tissue of *G. rigescens*. Based on the quantitative analysis, results of ANOVA and HCA indicated that the content of five iridoids existed a significant difference in root tissue of *G. rigescens* obtained from various locations. Results of CA and PCA showed that the climatic factor had a significant influence on the accumulation of LA, ST, GE, and SW in the same climatic zone. A high value of altitude and precipitation was unfavorable for the accumulation of these five iridoids. However, appropriate drought stress was meaningful for increasing the contents of iridoids. On the whole, our results have certain reference significance for the protection and cultivation of *G. rigescens*. These findings can provide scientific support for tracking geographical origin of *G. rigescens* and revealing the accumulation and changes of chemical components in different environments.

## Data Availability Statement 

All datasets generated for this study are included in the article/**Supplementary Material**.

## Author Contributions

LL conducted all laboratory experiments and drafted the article. Z-TZ was responsible for checking the grammar and revising this manuscript. F-RX guided the data analysis and partial interpretation of results. Y-ZW designed the study. All authors have contributed to the article and approved the submitted version.

## Funding

This work was supported by National Natural Science Foundation of China (81460581), the Key Project of Yunnan Provincial Natural Science Foundation (2017FA049) and Science and Technology Major Project of the Yunnan province (2017AB004).

## Conflict of Interest

The authors declare that the study was conducted in the absence of any commercial or financial relationships that could be construed as a potential conflict of interest.
