# Supplementary material for: Study on Quality Response to Environmental Factors and Geographical Traceability of Wild Gentiana rigescens Franch
Source: Front Plant Sci. 2020 Jul 22;11:1128. doi: 10.3389/fpls.2020.01128 (PMC7387691; doi:10.3389/fpls.2020.01128)
Supplement: Supplementary file 1 [file DataSheet_1.docx]

**Study on quality response to environmental factors and geographical traceability of** **wild *Gentiana rigescen*s Franch**

**Lu Liu^1, 2^, Zhi-tian Zuo^2^, Fu-rong Xu^1*^, Yuan-zhong Wang^2*^**

1 College of Traditional Chinese Medicine, Yunnan University of Chinese Medicine, Kunming 650500, China

2 Institute of Medicinal Plants, Yunnan Academy of Agricultural Sciences, Kunming 650200, China

***Correspondence:**

Dr Fu-Rong Xu, E-mail: xfrong99@163.com

Dr Yuan-Zhong Wang, E-mail: boletus@126.com

**Keywords:** ***Gentiana rigescens*, Environmental factors, Iridoid content, Quantitative determination,** **Fourier Transform Infrared, Quality control,** **Geographical traceability**

**Supplementary materials**

**
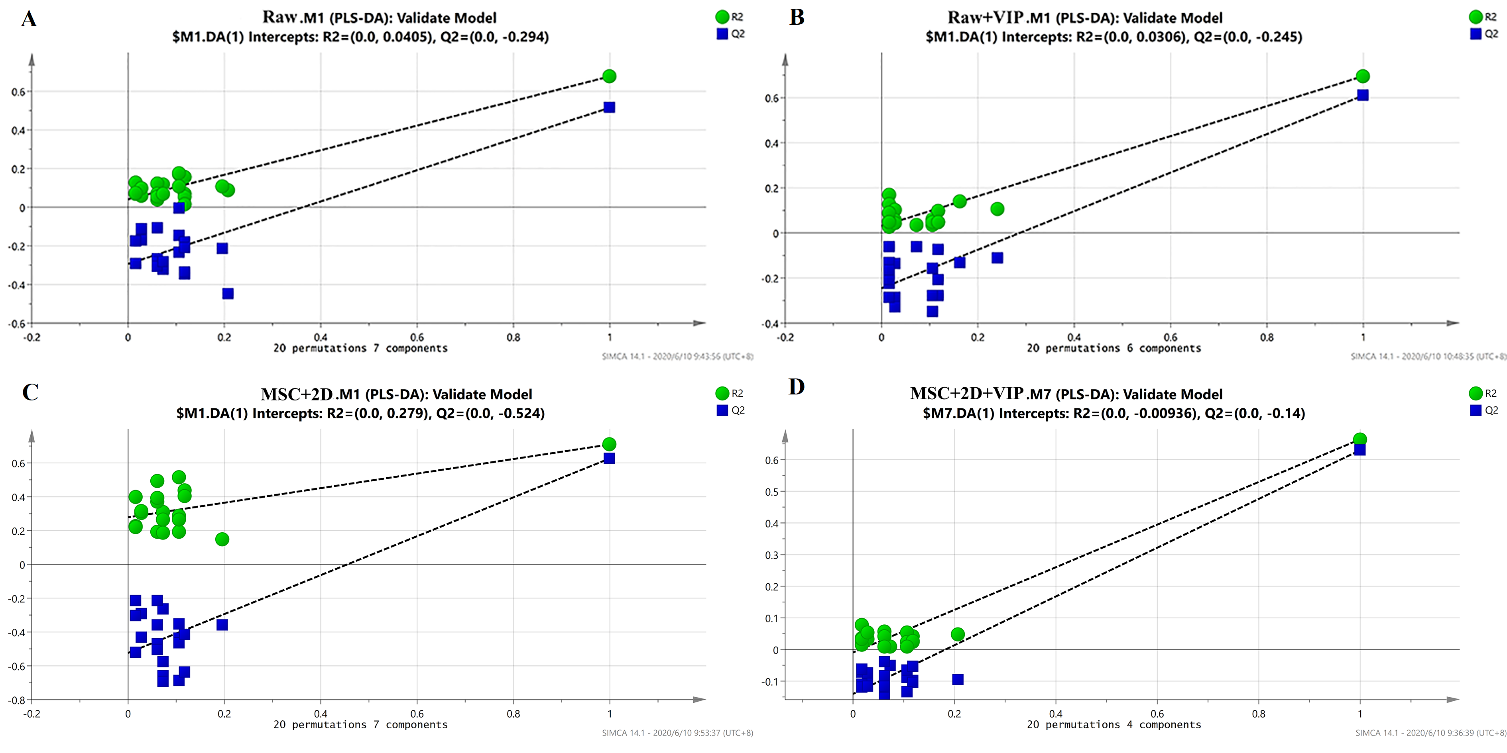
**

**Fig. S1** Permutation test results of the PLS-DA model


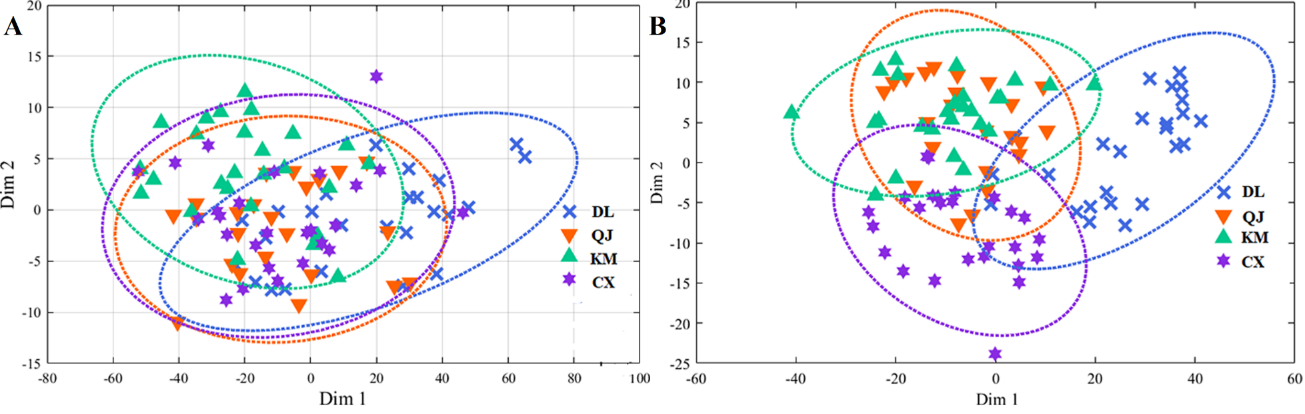


**Fig. S2** The scatter plot of partial least squares discriminant analysis (PLS-DA) model of *Gentiana rigescens* samples. A: The scatter plot of PLS-DA with original spectral data; B: The scatter plot of PLS-DA with preprocessed spectra data. DL: Dali; QJ: Qujing; KM: Kunming; CX: Chuxiong

**Table S1** The validation results of the method methodology

| Marked component | Regression equation (mg·mL^-1^) | R^2^ | Linearity range (mg·mL^-1^) | LOD (mg·mL^-1^) | LOQ (mg·mL^-1^) |
| --- | --- | --- | --- | --- | --- |
| loganic acid | Y=7115.1222X+24.8007 | 0.9999 | 0.0044-0.8001 | 0.0019 | 0.0062 |
| 6'-*O*-β-D-glucopyranosylgentiopicroside | Y=6704.3889X-30.9035 | 0.9999 | 0.0040-0.7998 | 0.0011 | 0.0035 |
| swertiamarin | Y=7976.8679X+25.1673 | 0.9999 | 0.0018-0.7683 | 0.0014 | 0.0046 |
| gentiopicroside | Y=5830.2818X+164.9953 | 0.9991 | 0.0498-3.6000 | 0.0347 | 0.1158 |
| sweroside | Y=4250.2396X-2.0569 | 0.9999 | 0.0018-0.7001 | 0.0018 | 0.0060 |

Notes: LOQ: the limits of quantification; LOD: the limits of detection.

**Table S2** The RSD value for precision, stability and repeatability in the methodology validation experiment

| Compound | Precision RSD (%) | | | | Stability RSD (%) | Repeatability RSD (%) |
| --- | --- | --- | --- | --- | --- | --- |
|  | inter-day | | | intra-day |  |  |
| loganic acid | 3.37 | 4.02 | 3.84 | 0.14 | 0.31 | 2.73 |
| 6'-*O*-β-D-glucopyranosylgentiopicroside | 4.29 | 4.02 | 2.98 | 2.47 | 4.18 | 2.82 |
| swertiamarin | 0.93 | 0.18 | 0.85 | 1.08 | 1.15 | 0.61 |
| gentiopicroside | 0.81 | 0.02 | 0.08 | 0.06 | 0.91 | 0.99 |
| sweroside | 3.06 | 1.48 | 3.15 | 1.14 | 1.25 | 4.49 |

**Table S3** The recovery test results of five iridoids in root tissues of *G. rigescens*

| Compound | Original amount (mg·mg^-1^) | Amount added (mg·mg^-1^) | Measured amount (mg·mg^-1^) | Recovery (%) | RSD (%) |
| --- | --- | --- | --- | --- | --- |
| loganic acid | 15.77 | 12.61 | 28.03 | 97.20 | 0.50 |
|  |  | 15.77 | 31.21 | 97.93 |  |
|  |  | 18.92 | 34.33 | 98.13 |  |
| 6'-*O*-β-D-glucopyranosylgentiopicroside | 0.84 | 0.67 | 1.51 | 98.97 | 0.46 |
|  |  | 0.84 | 1.67 | 98.10 |  |
|  |  | 1.01 | 1.84 | 98.33 |  |
| swertiamarin | 2.84 | 2.27 | 5.08 | 98.77 | 0.28 |
|  |  | 2.84 | 5.64 | 98.70 |  |
|  |  | 3.41 | 6.19 | 98.27 |  |
| gentiopicroside | 88.27 | 70.62 | 157.08 | 97.43 | 0.89 |
|  |  | 88.27 | 174.46 | 97.63 |  |
|  |  | 105.93 | 193.18 | 99.03 |  |
| sweroside | 3.33 | 2.67 | 5.94 | 97.93 | 1.18 |
|  |  | 3.33 | 6.67 | 100.10 |  |
|  |  | 4.00 | 7.26 | 98.27 |  |

**Table S4.** The confusion matrix of PLS-DA model established basing on different processed method.

| Model types |  | Training set | | | |  | Test set | | | |
| --- | --- | --- | --- | --- | --- | --- | --- | --- | --- | --- |
|  |  | O1 | O2 | O3 | O4 |  | O1 | O2 | O3 | O4 |
| Raw | O1 | 26 | 3 | 0 | 1 |  | 9 | 1 | 0 | 0 |
|  | O2 | 5 | 16 | 6 | 1 |  | 0 | 5 | 4 | 1 |
|  | O3 | 0 | 4 | 19 | 7 |  | 0 | 2 | 7 | 1 |
|  | O4 | 0 | 1 | 4 | 25 |  | 0 | 0 | 0 | 10 |
|  |  | O1 | O2 | O3 | O4 |  | O1 | O2 | O3 | O4 |
| Raw + VIP | O1 | 30 | 0 | 0 | 0 |  | 10 | 0 | 0 | 0 |
|  | O2 | 7 | 12 | 6 | 3 |  | 0 | 6 | 3 | 1 |
|  | O3 | 0 | 3 | 19 | 8 |  | 0 | 3 | 5 | 2 |
|  | O4 | 0 | 3 | 7 | 20 |  | 0 | 0 | 3 | 7 |
|  |  | O1 | O2 | O3 | O4 |  | O1 | O2 | O3 | O4 |
| MSC + 2D | O1 | 30 | 0 | 0 | 0 |  | 10 | 0 | 0 | 0 |
|  | O2 | 3 | 25 | 0 | 0 |  | 0 | 9 | 1 | 0 |
|  | O3 | 0 | 0 | 27 | 3 |  | 0 | 0 | 10 | 0 |
|  | O4 | 0 | 0 | 2 | 28 |  | 0 | 0 | 1 | 9 |
| MSC + 2D + VIP |  | O1 | O2 | O3 | O4 |  | O1 | O2 | O3 | O4 |
|  | O1 | 28 | 1 | 0 | 1 |  | 10 | 0 | 0 | 0 |
|  | O2 | 5 | 14 | 2 | 7 |  | 0 | 5 | 4 | 1 |
|  | O3 | 1 | 3 | 18 | 8 |  | 0 | 2 | 5 | 3 |
|  | O4 | 1 | 4 | 6 | 19 |  | 0 | 0 | 3 | 7 |

**Table S5.** The confusion matrix of SVM model established basing on different processed method.

| Model types |  | Test set | | | |
| --- | --- | --- | --- | --- | --- |
|  |  | O1 | O2 | O3 | O4 |
| Raw - GS | O1 | 10 | 0 | 0 | 0 |
|  | O2 | 1 | 6 | 3 | 0 |
|  | O3 | 0 | 0 | 7 | 3 |
|  | O4 | 0 | 0 | 3 | 7 |
|  |  | O1 | O2 | O3 | O4 |
| Raw - GA | O1 | 10 | 0 | 1 | 0 |
|  | O2 | 1 | 3 | 6 | 0 |
|  | O3 | 0 | 1 | 9 | 0 |
|  | O4 | 0 | 0 | 1 | 9 |
|  |  | O1 | O2 | O3 | O4 |
| MSC + 2D - GS | O1 | 10 | 0 | 0 | 0 |
|  | O2 | 0 | 10 | 0 | 0 |
|  | O3 | 0 | 0 | 10 | 0 |
|  | O4 | 0 | 0 | 0 | 10 |
| MSC + 2D - GA |  | O1 | O2 | O3 | O4 |
|  | O1 | 10 | 0 | 0 | 0 |
|  | O2 | 0 | 0 | 1 | 9 |
|  | O3 | 0 | 0 | 6 | 4 |
|  | O4 | 0 | 0 | 1 | 9 |
